# ENGAGING ASIAN AMERICAN FAMILY CAREGIVERS IN CLINICAL TRIALS: FINDINGS FROM A NATIONWIDE STUDY

**DOI:** 10.1093/geroni/igae098.2744

**Published:** 2024-12-31

**Authors:** Eve Rubovits, Anjali Yedavalli, Sabeen Sadruddin, Kavin Lavari, Raksha Mudar, Vania Leung, Minakshi Raj

**Affiliations:** University of Illinois Urbana-Champaign, Champaign, Illinois, United States; University of Illinois Urbana-Champaign, Champaign, Illinois, United States; University of Illinois Urbana-Champaign, Champaign, Illinois, United States; University of Illinois Urbana-Champaign, Champaign, Illinois, United States; University of Illinois, Champaign, Illinois, United States; University of Illinois Chicago, Chicago, Illinois, United States; University of Illinois Urbana-Champaign, Champaign, Illinois, United States

## Abstract

Ensuring representation of older racial and ethnic minority participants in clinical trials requires considering the family, community, and sociocultural context within which older adults make decisions about their healthcare and participation in trials. This study sought to evaluate Asian American caregivers’ (a) knowledge and experiences with clinical trials; (b) trusted and utilized sources of clinical trial information, (c) perceptions of ideal types of information to receive before enrolling their older relative in a clinical trial, and (d) concerns about their relative’s participation in clinical trials. We conducted a cross-sectional, online nationwide survey with Asian American family caregivers supporting an older relative between July 2022 and April 2023. Of all respondents (n=98), 62.2% reported knowing a little about clinical trials and 11.2% supported a relative who had been invited to participate in a clinical trial. Over one-third of respondents reported they most trust their relative’s healthcare provider as a source of information, and over half said they would use information from health organizations/groups or their relative’s provider for information. Respondents expressed wanting information about (a) the purpose, design, and components of the trial; (b) trial research ethics and safety; and (c) their responsibilities as the caregiver. Greater engagement between clinicians and family caregivers of culturally diverse older adults could help address concerns related to sociocultural barriers to participating in clinical trials. Providing key information to family caregivers in a comprehensive and accessible way without adding burden could help caregivers understand their responsibilities through the clinical trial process.

